# Complete mitochondrial genome and phylogenetic analysis of *Danio roseus* (Teleostei, Cypriniformes, Danionidae)

**DOI:** 10.1080/23802359.2022.2030257

**Published:** 2022-02-10

**Authors:** Lin Song, Xiao Jiang Chen, Xing Peng Han, Yi Wen Gu

**Affiliations:** Jiangsu Agri-Animal Husbandry Vocational College, Taizhou, Jiangsu Province, P. R. China

**Keywords:** Mitochondrial genome, *Danio roseus*, phylogenetic analysis, Danionidae

## Abstract

*Danio roseus*, collected from Gadu River in Yingjiang area, Yunnan Province, China, had been sequenced on Illumina HiSeq platform with 16523 bp in length, which included 37 genes encoding 13 proteins-coding genes (PCGs), 22 tRNAs, 2 rRNAs and a displacement loop region. The proportion of nucleotides in mitochondrial genome was T (28.7%), C (23.2%), A (32.3%), G (15.8%) with an AT bias of 61%. Maximum-Likelihood phylogenetic tree was reconstructed using concatenated mitochondrial protein-coding genes of *D. roseus* and other 12 fishes. The result of phylogenetic analysis supported the consanguineous relationship among *D. roseus*, *D. rerio, D. nigrofasciatus* and *D. dangila*.

The *Danio roseus* (Fang and Kottelat 2000) belongs to the subfamily Danioninae, family Danionidae, which is distributed in Yunnan Province of China; Lao People's Democratic Republic; Myanmar and Thailand. The characteristic description of the *D. roseus* is similar to that of *D. albolineatus* (Chu & Chen 1989). This project is the first to determine the mitochondrial genome sequence of *D. roseus*, which would be helpful to the study of its phylogenetic relationship and species identification.

The samples were obtained from Gadu River in Yingjiang area, Yunnan Province, China (24°60′81.86″ N, 97°66′19.38″E) on Jun 5th and stored at Aquatic Science and Technology Institution Herbarium (voucher ASTIH-21b0616d01, https://www.jsahvc.edu.cn/, Person in charge: Xiao Jiang CHEN, email: cq_cxj@126.com). Fresh samples were stored in dry ice and transported to Shanghai Genesky Biotechnologies Inc. The total genomic DNA was extracted from muscle tissues using Tguide Cell/tissue genomic DNA Extraction Kit (OSR-M401) (Tiangen, Beijing, China). The Illumina HiSeq 4000 Sequencing platform (Illumina, CA, USA) was used to perform the mitochondrial genome sequence. We used the software MetaSPAdes (Nurk et al. 2017) to assemble the sequence and annotated the genome with the MitoMaker software (Bernt et al. 2013). Phylogenetic tree was based on MEGA-X software (Tamura et al. 2013). The animal experimentswere approved by the Ethical Committee for Animal Experiments of Jiangsu Agri-animal Husbandry Vocational College and conducted in accordance with the Guidelines for Experimental Animals of the Ministry of Science and Technology (Beijing, China).

The complete mitochondrial genome of *D. roseus* was 16523 bp in length, which contained 37 genes encoding 13 proteins, 22 tRNAs, 2 rRNAs and 1 displacement loop region (D-loop) located between tRNA^Phe^ and tRNA^Pro^ with 913 bp in size. The proportion of nucleotides in mitochondrial genome was T (28.7%), C (23.2%), A (32.3%), G (15.8%) with an AT bias of 61%. Among all 37 genes, 28 genes were encoded on the H-strand while tRNA^Gln^, tRNA^Ala^, tRNA^Asn^, tRNA^Cys^, tRNA^Tyr^, tRNA^Ser(UCN)^, tRNA^Glu^, tRNA^Pro^ and *ND6* were encoded on L-strand. The characteristics of gene overlap and segregation were also observed in the mitochondrial genome of *D. roseus*. The interval length of 17 genes was 100 bp, ranging from 1 to 32 bp, and the overlap length of 9 genes was 28 bp, with overlapping base numbers between 1 and 7 bp (Table 1).

**Table 1.** Relevant features of *Danio roseus* mitochondrial genome.

	Position					Codon		
Gene	From	To	Nucleotide size (bp)	Amino acid	Space (+) overlap (−)	Initial	Terminal	Strand
tRNA^Phe^	1	69	69		0			H
12SrRNA	70	1023	954		0			H
tRNA^Val^	1026	1096	71		2			H
16SrRNA	1118	2760	1643		21			H
tRNA^Leu(UUR)^	2760	2834	75		−1			H
ND1	2836	3810	975	324	1	ATG	TAA	H
tRNA^Ile^	3815	3886	72		4			H
tRNA^Gln^	3885	3955	71		−2			L
tRNA^Met^	3957	4025	69		1			H
ND2	4026	5072	1047	348	0	ATG	TAG	H
tRNA^Trp^	5071	5140	70		−2			H
tRNA^Ala^	5143	5210	68		2			L
tRNA^Asn^	5212	5284	73		1			L
tRNA^Cys^	5317	5385	69		32			L
tRNA^Tyr^	5387	5457	71		1			L
CO I	5459	7009	1551	516	1	GTG	TAA	H
tRNA^Ser(UCN)^	7010	7080	71		0			L
tRNA^Asp^	7084	7152	69		3			H
CO II	7165	7855	691	230	12	ATG	T	H
tRNA^Lys^	7856	7929	74		0			H
ATPase8	7930	8094	165	54	0	ATG	TAA	H
ATPase6	8088	8771	684	227	−7	ATG	TAA	H
CO III	8771	9556	786	261	−1	ATG	TAA	H
tRNA^Gly^	9556	9624	69		−1			H
ND3	9625	9975	351	116	0	ATG	TAG	H
tRNA^Arg^	9974	10042	69		−2			H
ND4L	10043	10339	297	98	0	ATG	TAA	H
ND4	10333	11711	1379	459	−7	ATG	TA	H
tRNA^His^	11715	11784	70		3			H
tRNA^Ser(AGY)^	11785	11852	68		0			H
tRNA^Leu(CUN)^	11855	11927	73		2			H
ND5	11932	13731	1800	599	4	ATG	TAA	H
ND6	13727	14248	522	173	−5	ATG	AGG	L
tRNA^Glu^	14249	14317	69		0			L
Cyt b	14322	15462	1141	380	4	ATG	T	H
tRNA^Thr^	15463	15534	72		0			H
tRNA^Pro^	15541	15610	70		6			L
D-loop	15611	16523	913		0			H

Among 13 protein-coding genes (PCGs), the longest gene was *ND5* (1800 bp) and the shortest gene was *ATP8* (165 bp). Except that the *COI* gene took GTG as starting codon, the other 12 genes took ATG as starting codon. The PCGs (*ND1, COI, Atp8, Atp6, COIII, ND5,* and *ND4L*) took TAA as termination codon while two genes (*CO II, Cytb*) ended with incomplete termination codon (T) respectively, nevertheless the genes (*ND3, ND6*) ended with complete termination codon (TAG, AGG) respectively. The length of tRNA ranged from 68 to 75 bp. The 12S rRNA was 954 bp in length as well as 1643 bp of the 16S rRNA.

The sequence had been submitted to NCBI (NO. MZ853153). To find out the phylogenetic position of *D. roseus*, the mitochondrial genome sequences of 12 reported fishes were downloaded from NCBI GenBank, and 13 protein-coding genes (PCGs) of each fish were concatenated, respectively. Evolutionary analyses were conducted in MEGA X (Kumar et al. 2018) and the evolutionary history was inferred by using the Maximum Likelihood method and General Reversible Mitochondrial + Freq. model (Adachi and Hasegawa 1996). Initial tree(s) for the heuristic search were obtained automatically by applying Neighbor-Join and BioNJ algorithms to a matrix of pairwise distances estimated using the JTT + G + F model, with a bootstrap of 1000 replicates. The result of phylogenetic analysis showed *D. rerio* and *D. nigrofasciatus* grouped together, and they formed a sister-group with *D. roseus*, then the above three formed a sister-group with *D. dangila.* The analysis of phylogenetic tree supported the consanguineous relationship among *D. roseus*, *D. rerio, D. nigrofasciatus,* and *D. dangila* (Figure 1). Research on phylogeny of *Danio* suggested that there was a very close relationship among *D. albolineatus*, *D. rerio,* and *D. nigrofasciatus* (Fang 2003). The complete mitochondrial genome of *D. roseus* would be beneficial for resource conservation and phylogeny.

**Figure 1.**
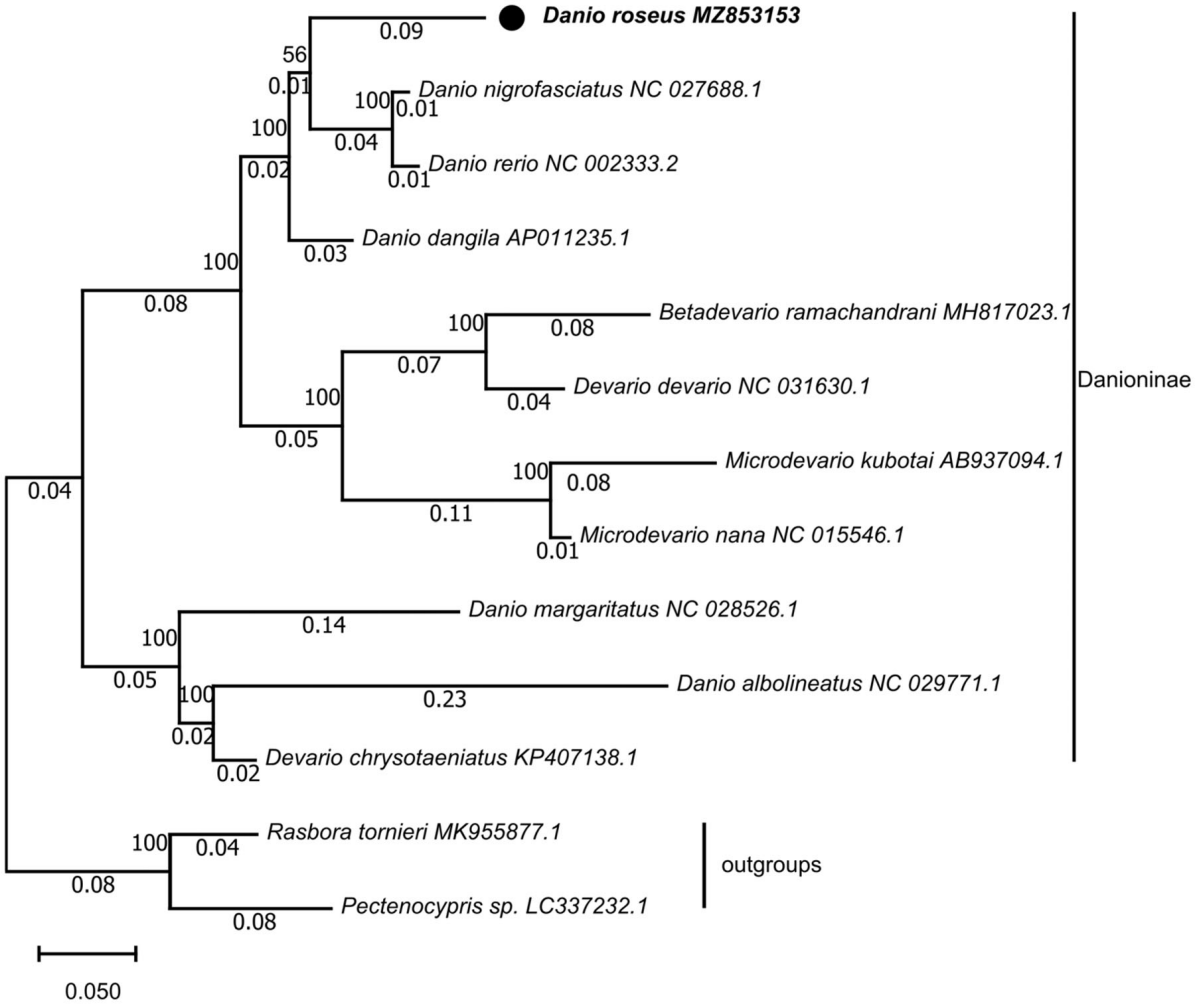
Maximum-likelihood (ML) phylogenetic tree reconstructed using concatenated mitochondrial protein-coding genes of *D. roseus* and other 12 fishes. *Pectenocypris sp.* and *Rasbora tornieri* were used as outgroups. Accession numbers are indicated after the species names. The tree topology was evaluated by 1000 bootstrap replicates. Bootstrap values at the nodes correspond to the support values for ML methods.

## Data Availability

The genome sequence data that support the findings of this study are openly available in GenBank of NCBI at (https://www.ncbi.nlm.nih.gov/) under the accession no. MZ853153 The associated "BioProject", "Bio-Sample" and "SRA" numbers are PRJNA769947, SAMN22183819, SRR16277915, respectively.

## References

[CIT0001] Adachi J, Hasegawa M. 1996. Model of amino acid substitution in proteins encoded by mitochondrial DNA. J Mol Evol. 42(4):459–468. 8642615 10.1007/BF02498640

[CIT0002] Bernt M, Donath A, Juhling F, Externbrink F, Florentz C, Fritzsch G, Pütz J, Middendorf M, Stadler PF. 2013. MITOS: improved de novo metazoan mitochondrial genome annotation. Mol Phylogenet Evol. 69(2):313–319. 22982435 10.1016/j.ympev.2012.08.023

[CIT0003] Chu XL, Chen YR. 1989. Ichthyology of Yunnan, China. Beijing, China: Science Press.

[CIT0004] Fang F, Kottelat M. 2000. *Danio roseus*, a new species from the Mekong basin in northeastern Thailand and northwestern Laos (Teleostei, Cyprinidae). Ichthyol Explor Fres. 11(2):149–154.

[CIT0005] Fang F. 2003. Phylogenetic analysis of the Asian Cyprinid genus Danio (Teleostei, Cyprinidae). Copeia. 2003(4):714–728.

[CIT0006] Kumar S, Stecher G, Li M, Knyaz C, Tamura K. 2018. MEGA X: molecular evolutionary genetics analysis across computing platforms. Mol Biol Evol. 35(6):1547–1549. 29722887 10.1093/molbev/msy096 PMC5967553

[CIT0007] Nurk S, Meleshko D, Korobeynikov A, Pevzner PA. 2017. MetaSPAdes: a new versatile metagenomic assembler. Genome Res. 27(5):824–834. 28298430 10.1101/gr.213959.116 PMC5411777

[CIT0008] Tamura K, Stecher G, Peterson D, Filipski A, Kumar S. 2013. MEGA6: molecular evolutionary genetics analysis version 6.0. Mol Biol Evol. 30(12):2725–2729. 24132122 10.1093/molbev/mst197 PMC3840312

